# Pediatrics

**Published:** 1898-10

**Authors:** Maud J. Frye

**Affiliations:** Clinical instructor in diseases of children, Medical Department, University of Buffalo


### PEDIATRICS.

Reported by MAUD J. FRYE, M. D.,
Clinical instructor in diseases of children, Medical Department, University of Buffalo.

TREATMENT OF CHOLERA INFANTUM.

POTTER (*Annals of Gynecology and Pediatry*, April, 1898,) advises the following treatment: Take away all food for twenty-four hours. Clear out the intestinal tract with calomel. Give larger quantities of a weak intestinal antiseptic solution by mouth, enough to keep the stomach well filled. Use the stomach-tube for its introduction if necessary. Irrigate the intestine with the same solution, using soft rubber catheter, No. 10 or 12, and a fountain syringe. If the stomach rejects the treatment, or there is persistent vomiting, let it rest for a time, in the meantime continuing the intestinal irrigations. If there is severe pain, use external applications or hypodermic injections of nonconstipating therapeutic agents. [Holt (*Diseases of Infancy and Childhood*), page 336, recommends morphine and atropine hypodermatically, not only for the relief of pain, but for the amelioration of the vomiting and purging, the nervous symptoms and the cardiac depression. To a child of one year 1-100 grain of morphine with 1-800 grain of atropine, may be given every hour if necessary. Morphine is contra-indicated if the purging is slight, or if there is drowsiness, stupor or relaxation.]

---

IRRIGATION OF THE BOWEL IN ENTEROCOLITIS.

Lichty (*Med. News*, April 16, 1898,) says: In cholera infantum and acute enterocolitis irrigation of the bowels with pure water is of such importance that, aside from proper dietetics, all other methods of treatment fall into insignificance. It is the most rational, and I think the most successful treatment which can be employed. It is very easily instituted. The nurse should be instructed to put on a rubber apron, and to take the child in her lap, laying it upon its abdomen, the lower end of the apron should be placed in a suitable receptacle to receive the wash water and excrement. The nozzle of a fountain syringe is then inserted in the child's anus, and the water allowed to flow continuously under slight pressure. Experiment has shown that there is no danger of rupturing the bowel, since the rectum in a child will expel the water after a certain pressure is attained. The child after two or three treatments of this kind will, in nearly every instance, be relieved.

---

SUBCUTANEOUS INJECTION OF SALT SOLUTION IN DIARRHEA.

BARBIES AND DERAYER (*Jahrbuch für Kinderheilkunde*) report the results of their observations, made on both well and sick infants, the object being to determine the physiological effects of a 7 per cent. sterilised salt solution.

From twenty to thirty grammes were injected at one time. The injections were followed by a period of reaction, lasting usually about seven hours. The temperature rose, the pulse increased in strength and volume, and the arterial fluxion also increased. All the functions of the body appeared to be stimulated. In collapse both respiration and circulation were favorably influenced. The temperature also became higher, the change being noticed about one-half hour after the injection. The rise in temperature varied from two to eight-tenths of a degree centigrade. At the end of five or six hours the temperature would again fall, but almost always remain from two to three-tenths higher than it was originally. The pulse usually increased about twenty beats per minute, and its further manifestations corresponded closely with the temperature. The authors believe the measure to be of service in cases of diarrhea with low temperature and collapse, or when collapse is threatened.—*Archives of Pediatrics.*

---

SUBCUTANEOUS INJECTIONS OF SERUM IN SUMMER DIARRHEA.

REICHNOCK (*Jahrbuch für Kinderheilkunde*). The indications for the injection of serum in diarrhea are excessive loss of fluid from the intestines and inability to take anything by the stomach. It is a means of giving nourishment to the patient with stomach and intestines at rest. The observations were made at Ranke's clinic, in Munich, in fifteen patients. Sterilised serum from healthy cows and horses was used. The cases selected were injected on the second or third day of the illness. They were greatly reduced in weight. Some were partially moribund. The ages of the patients varied from fourteen days to nine months. From ten to twenty cubic centimeters were injected at one time. No unpleasant symptoms followed in any of the cases. The beneficial effects were soon noticeable in an improvement in the general condition. In a few it was necessary to repeat the injections after twenty-four hours. The amount of albumin contained in the twenty centimeters, according to Hoppe-Seyler, is one to five grains. Four cases were fatal.—*Archives of Pediatrics.*

